# Probing the Solute-Solvent Interaction of an Azo-Bonded Prodrug in Neat and Binary Media: Combined Experimental and Computational Study

**DOI:** 10.1038/s41598-019-39028-1

**Published:** 2019-02-28

**Authors:** Abdulilah Dawoud Bani-Yaseen, Amina S. Al-Jaber, Heba M. Ali

**Affiliations:** Department of Chemistry & Earth Sciences, College of Arts & Science, Qatar University, Doha, State of Qatar

## Abstract

Preferential solvation has significant importance in interpreting the molecular physicochemical properties of wide spectrum of materials in solution. In this work, the solute-solvent interaction of pro-drug Sulfasalazine (SSZ) in neat and binary media was investigated experimentally and computationally. The solute-solvent interactions of interest were spectrophotometrically probed and computationally investigated for providing insights concerning the molecular aspects of SSZ:media interaction. Experimentally, the obtained results in 1,4-dioxane:water binary mixture demonstrated a dramatic non-linear changes in the spectral behavior of SSZ indicative of the dependency of its molecular behaviors on the compositions of the molecular microenvironment in the essence of solute-solvent interaction. Computationally, geometry optimization and simulation of the absorption spectra of SSZ in media of interest were performed employing DFT and TD-DFT methods, respectively, where the solvent effects on the absorption were examined implicitly using IEFPCM method. Obtained results revealed a nonpolar nature of the molecular orbitals that are directly involved in the SSZ:medium interaction. As in good correspondence with the experimental results, these simulations demonstrated that these orbitals are of non-polar nature and hence minimally affected by polarity of the media and in turn favoring the non-polar molecular environments. On the other hand, the molecular origin of SSZ:media interaction was demonstrated explicitly through complexation of SSZ with water molecules revealing a cooperative hydrogen bonding stabilization with an average length of 1.90 Å. The findings of this work demonstrate the significance of the preferential solvation and composition of the molecular microenvironment on the physicochemical properties of molecules of pharmaceutical importance.

## Introduction

The physicochemical behaviors of various materials of biomedical importance, including pharmaceuticals, can exhibit behaviors that are medium dependent^1–12^. This kind of amendment in physicochemical properties can be attributed to preferential solvation processes in solution. On the other hand, the solvation progression of soluble molecules in media of interests is selectively governed by various types of noncovalent solute-solvent interactions. These interactions are categorized by intermolecular forces that can lead to modification of the physiochemical nature of a solute, which in turn may give rise to medium-dependent molecular properties^13–19^. These intermolecular interactions are of noncovalent nature and can include, but not limited to, hydrogen bonds and electrostatic attractions. Accordingly, it has been demonstrated that the physicochemical behavior of a drug can be influenced by its molecular environments. For example, Ohno *et al*. demonstrated via utilizing quantum mechanics calculations the effect of solvent hydration on the reactivity of ribonuclease T1 enzyme by tuning its activity and retaining its native structure^20^. Recently, Balius *et al*. reported on the role of solvation in aqueous media on structure-based ligand discovery of proteins of interest^21^. They demonstrated the significance of how the water molecules can crucially influence the protein–ligand binding.

**Figure 1:**
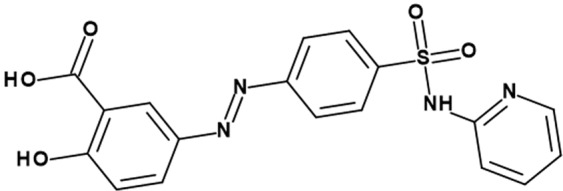
Chemical structure of SSZ.

It is noteworthy mentioning that the solvation of substances of pharmaceutical interests can occur in both neat and binary media. As water is the biological solvent, prospective aspects are more frequently emphasized on aqueous media^1,2,6^. However, aqueous binary media are imperative to be considered as well, where a combination of water and organic solvent, such as 1,4-dioxane and acetonitrile, is utilized for studying the physicochemical properties of pharmaceuticals^22–33^. Nevertheless, most of these reported studies have considered mainly the examination of the solubility of these materials in the media of interest without considering the effect of intermolecular interactions of the media with the solute. Thus, further investigations concerning the solute-solvent interactions of pharmaceuticals are still necessary in this regard. To this end, the solute-solvent interactions and their corresponding effects have been investigated utilizing various experimental and computational methods^34–41^. Indeed, combination of experimental and computational represents an inimitable opportunity for observing such kind of interactions, where the experimental results can be molecularly interpreted using various computational approaches^36^. Thus, in view of the potential influence of several media properties on the molecular properties of pharmaceutical materials, such as Sulfasalazine (SSZ), there is a necessity toward gaining insights regarding such influences in terms of molecular interactions. SSZ, 2-hydroxy-5-[(E)-2-{4-[(pyridin-2-yl)sulfamoyl]phenyl}diazen-1-yl]benzoic acid, is a prodrug that is often prescribed for treatment of inflammatory bowel disease^42,43^. The structure of SSZ is shown in Fig. 1.

As can be noticed in Fig. 1, SSZ represents a unique property conferred upon it by the variety of functional groups that can potentially facilitate the noncovalent solute-solvent interactions with components present within its molecular environments. In this work, we aim at providing insights into the molecular solute-solvent interactions of SSZ with its media. The UV-Vis absorption spectroscopy was used to probe experimentally the occurrence of this interaction followed by DFT/TD-DFT calculations for interpreting the molecular behavior of this kind of interaction.

## Methods

### Experimental procedure and method

SSZ and all neat solvents of spectroscopic grades were purchased from Sigma-Aldrich; all chemicals were used as received. The UV-Vis absorption spectra of SSZ were measured in neat solvents and 1,4-dioxane: water binary mixtures of v:v ratio within the range 0–100%. SSZ stock solution was prepared in methanol with concentration of 3.06 × 10^−4^ M. The preparation was conceded out by transferring a 0.6 mL of stock solution, then evaporating the methanol under medium pressure at room temperature, followed by re-dissolving the residue of SSZ in 10 mL of the medium of interest. The UV–Vis absorption spectra in the media of interest were measured using Agilent double beam spectrophotometer in quartz cells.

### Computational methods

All calculations were conducted using the *Gaussian 09* software package^44^. Structure of SSZ was optimized followed by frequency calculations employing the density functional theory (DFT) with B3LYP/6-31 + G(d) basis sets. The solvent effect was examined implicitly employing the integral equation formalism polarizable continuum model (IEF-PCM)^45^. The UV-Vis absorption spectra of SSZ were simulated employing the Time-dependent DFT (TD-DFT) with two basis sets; namely, B3LYP/6-31 + G(d) and CAM-B3LYP/6-31 + G(d). Simulations were conducted using the optimized geometry as an input. Explicit solvent effect was examined using water and 1,4-dioxane molecules for complexation with SSZ at the same level of theory.

## Results and Discussion

**Figure 2:**
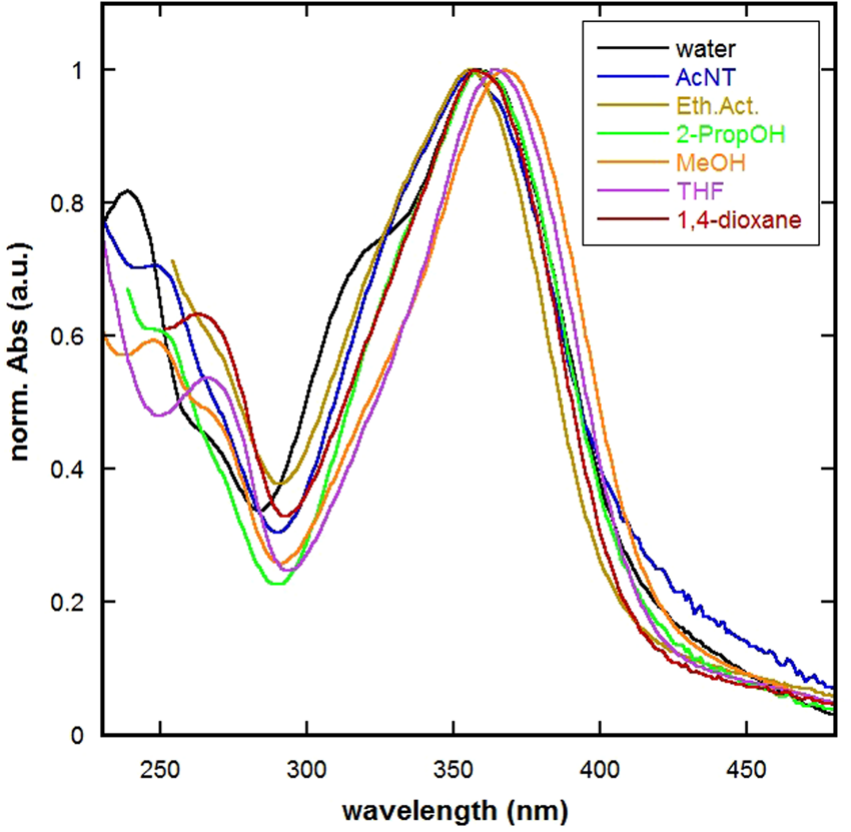
Normalized absorption spectra of SSZ in selected neat solvents.

The absorption spectra of SSZ in selected neat solvents of different polarity and bearing various hydrogen bonding abilities were measured. Normalized absorption spectra at unity in selected solvents are displayed in Fig. 2. As can be noted, the absorption spectrum of SSZ exhibits main absorption bands within the range of 230–450 nm, where its position obviously varies depending on the nature of the medium. In particular, the illustrations in Fig. 2 depict SSZ absorption spectra at maximum absorption bands of ~265 (λ_max1_) and 360 nm (λ_max2_) that can be due to π−π* electronic transition as demonstrated by the TD-DFT calculations. Furthermore, SSZ spectrum exhibited a notable shoulder at a wavelength of ~320 nm in aqueous media; however, this band did not appear in other media. As this might be attributed to either overlap with other bands or retarded electronic transitions, further analyses were necessitated as described in the computational part of this study. Upon further examination of Fig. 2, one can notice that the absorption spectra of SSZ exhibit media-dependent features, where this dependency can be rationalized for probing the SSZ-media interactions. In addition, it is interesting to note that both of λ_max_’s exhibit solvent dependency. However, the significant difference is the disappearance of the shoulder in non-aqueous media as the spectra pass from a non-polar to a polar microenvironment, such as 1,4-dioxane and water respectively. This spectral behavior can be attributed to the fact that SSZ’s absorption spectrum appears at different wavelength as solvent polarity varies indicative of general type of interaction.

Considering the main band at λ_max2_ (360 nm), shifts in a hypsochromic manner is noted. Taking into consideration the solvent effects on the ground and excited states of SSZ, this spectral behavior is influenced by increasing the solvent polarity indicative of relatively higher dipole moments in the ground state compare to the excited state. Indeed, in addition to media polarity, upon taking other media parameters into consideration, specific interactions including hydrogen bonding cannot be neglected. This is in the sense that such noncovalent interactions play a key role in influencing the magnitude of each other within the geometry of SSZ. The vital physical parameters of solvents to be considered herein include refractive index, orientation polarizability, solvent electric permittivity, and Kamlet-Taft solvent parameters. Initially, to understand molecularly the solvatochromic behavior of SSZ and their correlation with different solvent’s parameters, the properties of λ_max2_ in particular and electronic absorption spectrums were correlated with various pertinent scales of polarity. In fact, it is more reasonable to considered the combined effect of solvent’s refractive index and electric permittivity as presented by orientation polarizability (∆ƒ (ε, n)), which can expressed as:

1 $${\rm{\Delta }}f=\frac{{\rm{\varepsilon }}-1}{2{\rm{\varepsilon }}+1}-\frac{{n}^{2}-1}{2{n}^{2}+1}$$

where, ε is solvent’s electric permittivity and n is its refractive index. The plot of λ_max2_ as presented by wavenumber as a function of ∆ƒ is shown in Figure S1. As can be noted, the correlation between absorption wavenumber and polarizability orientation of SSZ in different solvents of various polarities has inferred that there is a non-linear correlation between ∆ƒ and λ_max2_ over the range of tested solvents. This observation may be inferred as the existence of specific intermolecular interactions in addition to the polarizability effect; this mainly includes the effect of hydrogen bonding. Henceforth, the existence of specific interaction can be practically examined employing the Kamlet-Taft approach. Examining the molecular structure of SSZ, one can notice that SSZ can intermolecularly interact with the medium through donating and accepting hydrogen bonding. Importantly, the Kamlet-Taft method takes into consideration the combined effect of the solvent’s parameters through conducting a multi-linear-regression-analysis (MLRA) according to eq. 2:

2 $${{\rm{\lambda }}}_{{\rm{\max }}}={{\rm{\lambda }}}_{{\rm{o}}}+{\rm{a}}{\rm{\alpha }}+{\rm{b}}{\rm{\beta }}+{c{\rm{\pi }}}^{\ast }$$

where, λ_max_ = maximum wavelength, λ_o_ = regression intercept/gaseous phase, α = hydrogen bond donor acidity (HBD), β = hydrogen bond donor basicity (HBA), π^*^ = index of solvent’s dipolarizability, a, b, and c are independent constants, whose sign and magnitude portray the extent of corresponding solvent-solute interactions and their effect on the absorption maximum wavelength. The Kamlet-Taft parameters of examined solvents are compiled in Table S.1; this includes β, α, and π*. Based on the MLRA of Kamlet-Taft method, this analytical approach demonstrates that the structure of SSZ constitutes different functional polar groups, with the ability of facilitating the hydrogen bondings in patterns that promote solute-solvent interactions, which in turn can be specific in nature. An MLRA test on neat solvents yielded the following correlation as noted in eq. 3:

3 $${{\rm{\lambda }}}_{{\rm{\max }}2}=(324\pm 14.5)+(45\pm 17){{\rm{\pi }}}^{\ast }-(11\pm 7){\rm{\alpha }}+(30\pm 12){\rm{\beta }}$$

The MLRA results revealed a fair level of acceptancy of correlation (R = 0.83) indicative of a relatively complexed correlations that in turn necessitates applying further analysis. Interestingly, the high tendency of SSZ to form strong hydrogen bonding with the media of interest can be elucidated through examining the effect of strong hydrogen bond acceptor, such as water, on the absorption spectrum of SSZ using aqueous binary media; namely 1,4-dioxane:water binary mixture, as illustrated below.

**Figure 3:**
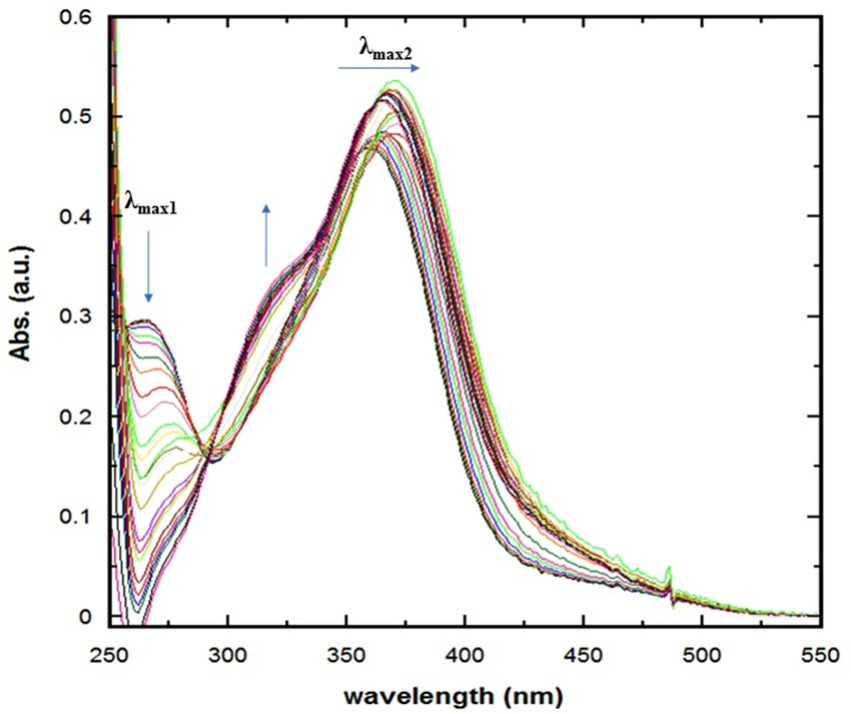
Absorption spectra of SSZ in 1,4-dioxane:water binary mixture of varying composition.

Recently, one can notice a growing interest in employing binary solution systems for investigating the physicochemical properties of various materials. The molecular behavior of solutes can exhibit a molecular behavior in the binary systems that is relatively different compared with the individual neat solutions. Hence, employing such binary systems can offer biologically mimicked molecular environment, which in turn can provide enhanced and relatively more informative systematic examination of the physicochemical properties for the solutes of interests compared with neat solvents. Among the most popular binary systems in this regard is 1,4-dioxane:water mixture^24,46–52^. Such popularity can be attributed to the similarity of these two solvents in terms of viscosity and density in addition to their miscibility of all fractions despite the large difference in their polarity. To this end, the absorption spectra of SSZ in 1,4-dioxane:water binary media of variable volumetric fractions (v_diox_:v_w_) are displayed in Fig. 3. The arrows indicate the spectral behavior with increasing water content in the binary system. Starting with neat 1,4-dioxane and then increasing the water content, as can be noted, the absorption peak at λ_max1_ and shoulder at λ_320_ start to fade and grow, respectively, with isopiestic point at λ of 290 nm in all ratios of the binary mixtures. Concerning the λ_max1_, as expected, on can notice that the effect of the nature of 1,4-dioxane started to overcome the spectral features of SSZ, which in turn retards further conclusion to be reached at this range. In addition, the shoulder at λ_320_ reached a maximum growth with v_w_ of approximately 70%. On the other hand, for the main absorption peak at λ_max2_, SSZ absorption spectrum exhibited λ_max2_ of 358 and 360 nm in neat 1,4-dioxane and water, respectively. Further examination of Fig. 3, one can notice that the water content did not only influenced λ_max2_, but also the absorbance value Abs_max_ at λ_max2_.

**Figure 4:**
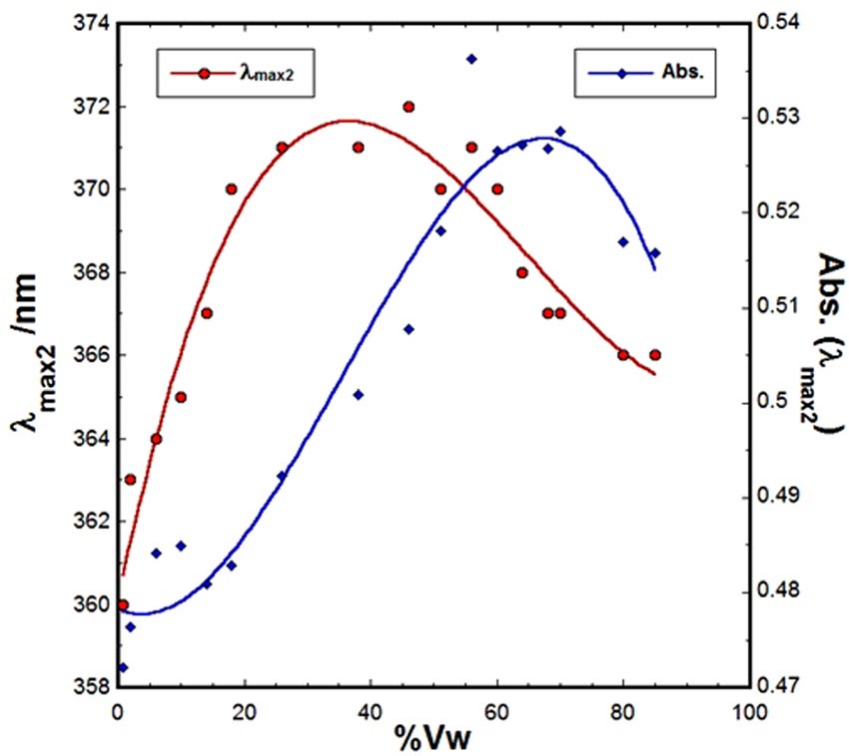
Plots of spectral properties of SSZ (λ_max2_ and Abs._max_) as functions of water component (v_w_).

Quantitatively, the spectral behavior of SSZ in the binary system in terms of λ_max2_ and Abs_max_ as functions of v_w_ are displayed in Fig. 4. Interestingly, as can be noted in Fig. 4, λ_max2_ exhibited a dramatic bathochromic shift with the increase of water content in the binary mixture to reach a maximum value of 271 nm at v_w_ of 35%. However, with increasing water content, λ_max2_ started to show hypsochromic shift behavior to reach a new final value of 365 at high v_w_. On the other hand, for Abs_max_, no change was observed for solutions of v_w_ ≤ 20%, yet a bathochromic shift in λ_max2_ of ~9 nm was observed. A maximum change in Abs_max_ is observed at v_w_ of 70%. These dramatic changes in the spectral behavior of SSZ are indicative of the dependency of its molecular behaviors not only on the type of media, but also on the compositions of its molecular microenvironment in the essence of solute-solvent interaction.

Importantly, it is must be mentioned that the nonlinearity of the physicochemical properties of 1,4-dioxane:water binary mixtures have been reported by Takamuku *et al*.^53^. They reported three microscopic sub-systems with respect to the molar fraction of 1,4-dioxane (x_diox_). For solutions of compositions of x_diox_ ≤ 0.1, x_diox_ ≥ 0.3, and 0.1 ≤ x_diox_ ≤ 0.3, three sub-systems were distinguished; namely, water hydrogen-bonded network, 1,4-dioxane:water hydrogen-bonded network dominated by 1,4-dioxane, and 1,4-dioxane:water binary clusters, respectively. Hence, the non-linear correlation between the spectral behavior of SSZ and the composition of 1,4-dioxane:water binary mixtures is in good correspondence with these microsystems and in good agreement with previously reported results concerning the properties of 1,4-dioxane:water binary mixtures.

**Figure 5:**
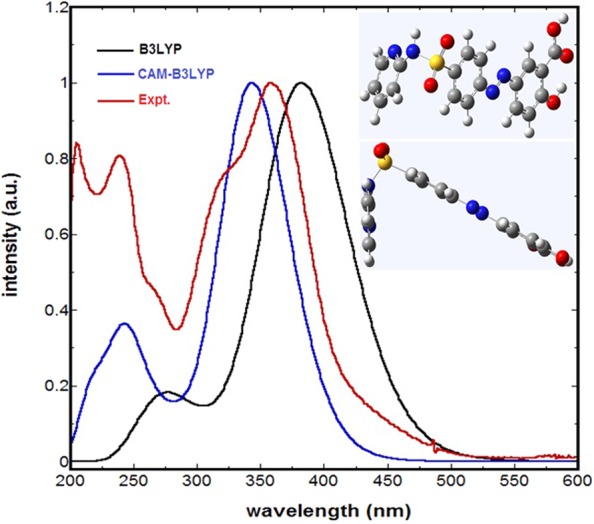
Normalized experimental and simulated absorption spectra of SSZ in water; inset: side and top views of optimized geometry of SSZ.

As can be noted from the aforementioned experimental results, computational invistigations are necessary for elucidating the molecular origin of SSA:media interaction. In this regard, DFT and TD-DFT calculations were performed in the essence of providing molecular interpretation concerning the SSZ-medium interactions. The initial geometry optimization of SSZ was performed in vacuum using the DFT method with B3LYP functional and 6–31 G + (d) basis set. The optimized geometry in vacuum was then used as entry for optimization in the media of interest employing the same level of theory and IEFPCM solvation approach. The frequency calculations of all optimized geometries confirmed that these geometries are of minimal energy. Geometry optimization calculation revealed dipole moment (μ) of 5.7 and 7.0 Debye in 1,4-dioxane and water, respectively, indicative of the slight increase in polarization of SSZ in aqueous media compared with 1,4-dioxane. TD-DFT calculations were conducted using the optimized geometry as initial entry for the calculations in the corresponding media. To this end, it must be mentioned that employing the most appropriate DFT functional to be considered for simulating the absorption spectra of SSZ in various media is judged based on matching with the experimental spectrum. In this regard, two DFT functionals were tested with the same basis set; namely, B3LYP and CAM- B3LYP. Simulations were performed for the first twelve transition bands. The simulated and experimental absorption spectra of SSZ in water are shown in Fig. 5. The inset shows the side and top views of the optimized geometry of SSZ used for generating the simulated spectra.

**Figure 6:**
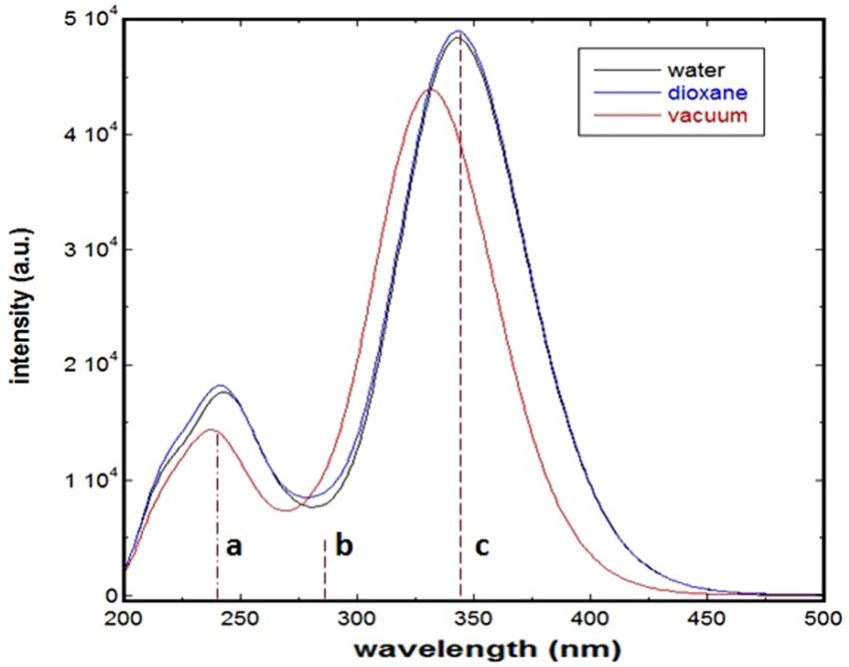
Simulated absorption spectra of SSZ in vacuum, water, and 1,4-dioxane.

Examining Fig. 5, as compared with the experimental spectrum, one can notice that the B3LYP and CAM- B3LYP over-estimated and under-estimated the electronic transitions, respectively, with ∆λ of approximately 10 and 26 nm, respectively, with respect to the experimental λ_max2_. Further analysis was performed employing the same functionals for other solvents; this includes methanol, 1,4-dioxane, and acetonitrile. Simulated absorption spectra of SSZ in these selected solvents are displayed the supplementary information; see Figure S3. As can be noted in Figure S3, a systematic underestimation and overestimation of λ_max2_ is observed using B3LYP and CAM- B3LYP functionals, respectively, with no discrepancies among solvent. However, for the rest of the spectrum, employing the CAM- B3LYP functionals has successfully re-produced a spectrum that is in excellent agreement with the experimental one in terms of spectral shape. This can be noticed in particular for λ_max1_ and the shape of the spectrum at λ ≤ 300 nm. Hence, CAM- B3LYP functionals was employed for other media as well. Simulated spectra of SSZ in vacuum, 1,4-dioxane, and water are shown in Fig. 6. The vertical lines represent the major electronic transitions that exhibit oscillation strength (ƒ) ≥ 0.1. Likewise, the same transitions were observed in all media. However, the simulated spectra in 1,4-dioxane and water did not exhibit media dependent for λ_max2_, where a value of 343.5 nm was obtained for both media with minimal discrepancy in oscillation strength ƒ of 1.203 and 1.190, respectively. Whereas for λ_max1_, a bathochromic shift of ∆λ of ~1.5 nm was observed in aqueous media, which in turn is in fair correspondence with the experimental results. For the third electronic transition band, labeled as b in Fig. 6, hypsochromic shift of ∆λ of ~2.0 nm was observed in aqueous media indicative of fair separation from the main band c. This separation between bands b and c exhibited an increase of only 2.0 nm in aqueous media, yet more separation permits the appearance of the shoulder that is qualitatively in good correspondence with the experimental results. However, one can confer the unsatisfactory behavior of the simulated systems in terms of interpreting the experimental results; indeed, this observation can be attributed to the general nature of implicitly of the simulated medium that does not take into consideration specific types of intermolecular interactions including HB.

**Figure 7:**
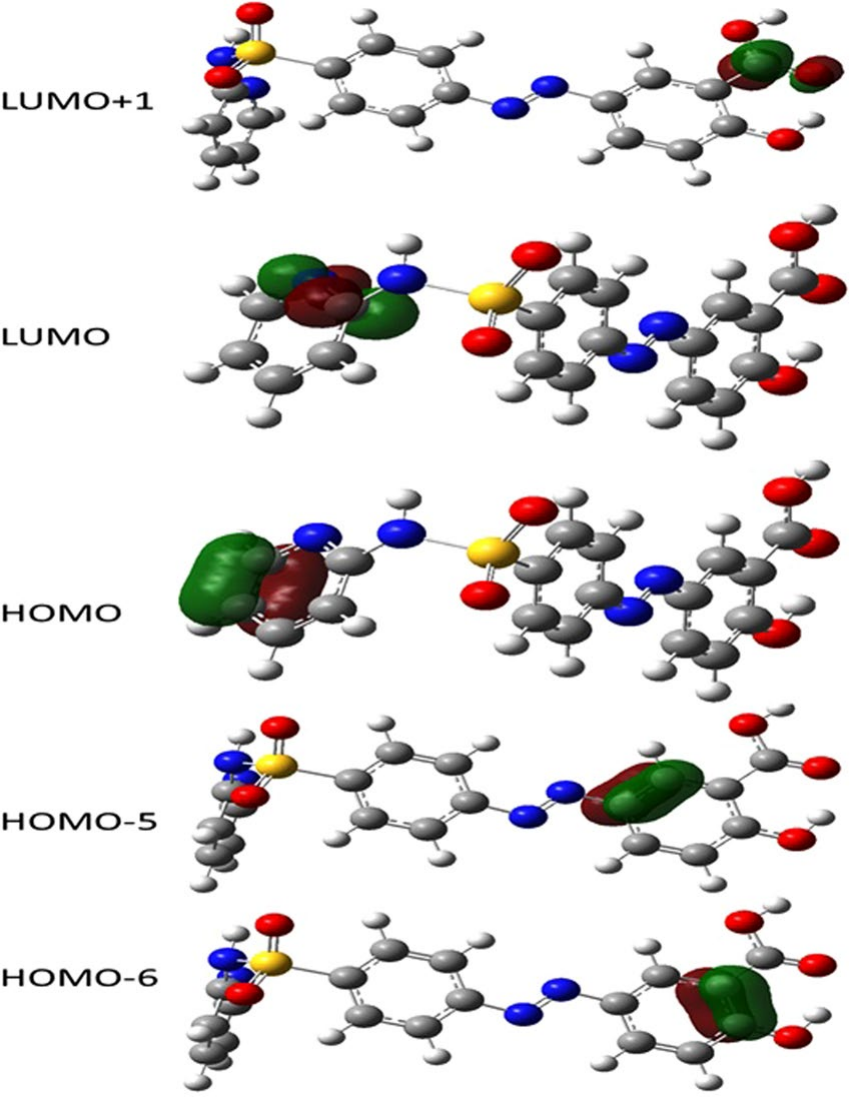
Molecular orbitals of SSZ involved in the electronic transitions of the main three bands in the absorption spectrum.

Interestingly, one can notice that the main three electronic transitions observed experimentally in neat aqueous media, namely two peaks and shoulder in particular, were reflected as only two peaks in the simulated spectrum, which in turn may be attributed to insufficient polarization of SSZ in the corresponding examined medium employing the implicit solvation model. Nevertheless, although the shoulder did not appear in the simulated spectrum in aqueous medium, the electronic transition exhibited an oscillation strength of approximately 0.12, which in turn is worth of further consideration. In addition, it can be noticed that the experimental shoulder at λ_320_ did now show up computationally in all media. Considering the experimental disappearance of this shoulder in non-aqueous media, this might be attributed to specific solute-solvent interactions involving specific molecular orbitals (MOs) of SSZ, which in turn requires performing more computational analyses concerning the MOs of SSZ involved in the electronic transitions of interest. Correspondingly, the MOs of interest of SSZ were simulated for the optimized geometries in all media; see Fig. 7. Based on TD-DFT simulations, these three electronic bands can all be attributed to π → π^*^ electronic transitions. For the main band (c), HOMO → LUMO transition is the only contribution to the main band of λ_max2_. As shown in Fig. 7, both the HOMO and LUMO are distributed over the pyridine moiety of SSZ, which are part of a character that is aromatic in nature, which in turn makes them less subjective to be influenced by the interaction with molecular media. This is in good agreement with the minimal shift in λ_max2_ observed experimentally in water compared with 1,4-dioxane. For the second band, b, the main contributions are HOMO-6 → LUMO + 1 and HOMO → LUMO + 1 electronic transitions. The LUMO + 1 is the π^*^ of C = O of the carboxyl group. We believe that the experimental appearance of this band as shoulder in aqueous band might be attributed to the increase in the population of this state as it is more stabilized in polar media. For the first band, a, the main contributions are HOMO-6 → LUMO + 1, HOMO-5 → LUMO, and HOMO → LUMO + 1 electronic transitions. Likewise, the aromatic nature of the main MO that contributes in this transition, namely HOMO-5, is one of the π MO of the benzene ring of salicylic acid moiety, which in turn disfavors the polar molecular environments and hence less populations might be observed in aqueous media compared with 1,4-dioxane.

**Figure 8:**
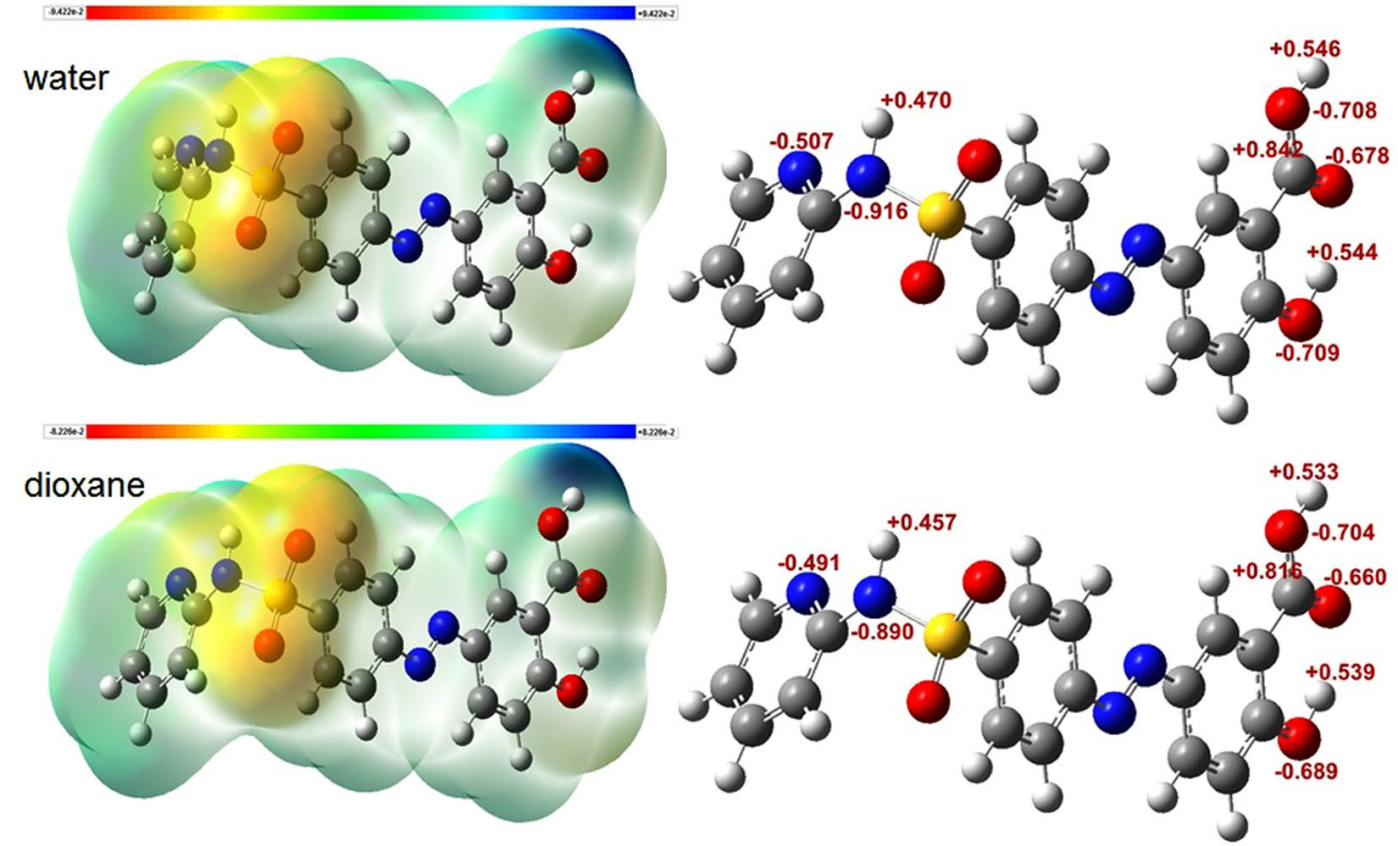
Molecular properties of SSZ in water and 1,4-dioxane: (left) the electrostatic potential surfaces, (right) NBO charges.

As noted above, most of the MOs involved in the electronic transitions as the main contributors in the three bands of SSZ absorption spectrum are of non-polar nature and hence favoring the non-polar molecular environment. Thus, we further attempted to provide more interpretation regarding the polarity effect on SSZ-medium interactions. As stated earlier, the geometry optimization revealed an increase in the diploe moment (∆μ) of only 1.3 Debye in water compared with 1,4-dioxane. These effects can be viewed with respect to the electrostatic potential surfaces (EPS) of the molecule, and localized charge effect of SSZ atoms (NBO charges), as illustrated in Fig. 8.

**Figure 9:**
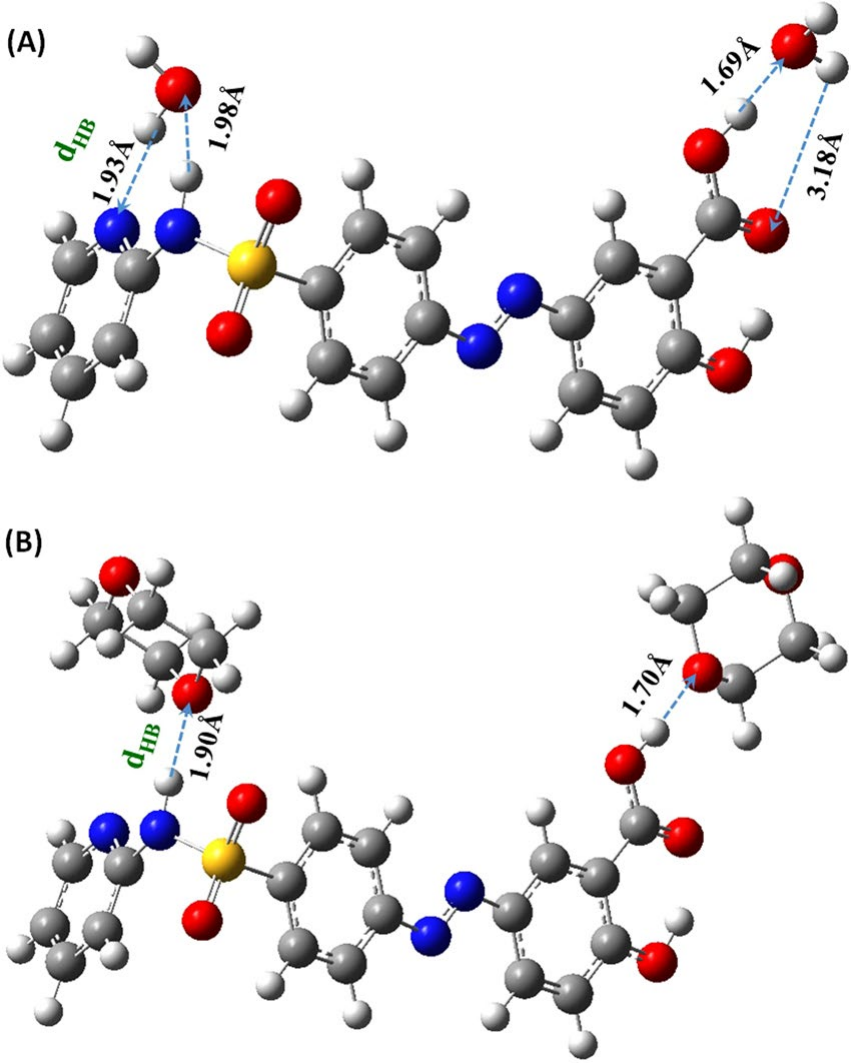
Optimized geometry of hydrogen-bonded complexes of SSZ:2H_2_O (**A**), and SSZ:2 1,4-Dioxane (**B**).

As can be noted in Fig. 8-left, EPS simulations revealed an increase of approximately 15% in the charge distribution across the molecule in aqueous media compared with 1,4-dioxane, which can be mainly noted for the change in the bluish color of the carboxyl group of the salicylic acid moiety. This is indicative for potentially more specific solute-solvent interaction; namely, hydrogen bonding. On the other hand, the NBO calculations revealed the atomic charges of all atoms of SSZ molecule; see Fig. 8-right. Correspondingly, we focused on functional groups that are subjective for potential solute-solvent interactions and can be involved in the electronic transitions of the main three bands of SSZ absorption spectrum; this includes the azo, carboxyl, and hydroxyl of the salicylic acid moiety, and the amide group of the pyridine moiety. Hence, we observed an increase in the polarization of all bonds in aqueous media with a charge difference of an average of approximately 0.02. This minimal change in bond polarization is in good correspondence with the dipole moment and EPS calculations. Furthermore, Figs 7 and 8 revealed that the moieties of SSZ that can be utilized for specific intermolecular interactions with solvent molecules, which in turn may have consequential effects on the absorption spectra of SSZ. As proof of principle, a combination of explicit and implicit solvent effects of water and 1,4-dioxane was examined through complexation of two solvent molecules with one SSZ molecule employing DFT/CAM-B3LYP/6-31 G + (d) method in combination with IEFPCM solvation approach. Figure 9 illustrates the optimized geometry of the corresponding HB complexes in water and 1,4-dioxane, respectively. Importantly, the molecular origin of solvent effect is explicitly demonstrated through cooperative hydrogen bonding of SSZ with solvent molecules. For SSZ:H_2_O complex, it is noteworthy mentioning that the with hydrogen bond length (d_HB_) of approximately 1.90 Å was obtained for only the explicit solvation effect.

However, notable increase to 3.18 Å in the d_HB_ involving the carboxyl group upon employing the combination with implicit solvent effect of the IEFPCM method with minimal effect on the other HBs. It must be mentioned that water molecules behave in this scenario as cooperative HB donor and acceptor as illustrated by the arrows in Fig. 9. On the other hand, 1,4-dioxane behaves only as an HB acceptor. An average d_HB_ of approximately 1.80 Å is obtained between SSZ and 1,4-dioxane molecules. Interestingly, the cooperativity of hydrogen bonding of SSZ:2H_2_O complex, where SSZ can synchronizingly act as hydrogen donor and acceptor, is in good agreement with the experimental results as revealed by the MLRA of the spectral behavior of SSZ in different solvents. The explicit-implicit solvent combined effect of water and 1,4-dioxane on the absorption spectra of SSZ was examined; obtained results are displayed in Figure S4. Comparing the two spectra, one can notice an insignificant effect of solvent on λ_max2_ with obtained values of 344.4 and 345.8 nm in water and 1,4-dioxane, respectively, which in turn is in fair agreement with the experimental results in terms of the red shift of 2 nm observed in 1,4-dioxane compared with water. However, the shoulder at λ_320_, which is observed experimentally for SSZ in aqueous medium, was not observed computationally. Furthermore, the λ_max1_ exhibited no solvent dependency. In addition, a slight increase of ~1% in the ƒ value was observed in both solvents for the main band λ_max2_ compared with the implicit solvation effect.

## Conclusions

The solute-solvent interaction of SSZ was successfully demonstrated experimentally in neat and binary 1,4-dioxane:water mixtures. The electronic UV-Vis absorption band of SSZ that matches to the transitions in the π − π* illustrated notable solvatochromic shifts with changes in the absorption spectrum indicative of the influence exerted by solvent-solute interactions on the corresponding electronic transitions. As in good agreement with the nature of 1,4-dioxane:water binary mixture, a non-linear correlation was demonstrated for the spectral behavior of SSZ in the mixture indicative of adaptation of SSZ to the nature of its local molecular microenvironment. Computationally, the absorption spectra of SSZ were simulated successfully through TD-DFT calculations taking into consideration the implicit and explicit solvation approaches. Although these calculations successfully reproduced the absorption spectra of SSZ in terms of main transition bands, insignificant spectral shift was observed employing the implicit and explicit solvent effects. In addition, it has been demonstrated that the MOs involved in these electronic transitions are of non-polar nature indicating the fair favoritism of the corresponding non-polar molecular environment. The EPS and NBO calculations, as in good correspondence with the TD-DFT calculations, confirmed the fair effect of the polarity of the molecular microenvironment on charge distribution across the molecule and the localized atomic charges, respectively, with more charge separation is observed in aqueous media. Furthermore, SSZ can molecularly interact with solvent molecules through strong cooperative hydrogen bonding as explicitly demonstrated in water and 1,4-dioxane. These observations can in turn be utilized for interpreting the physicochemical behavior of SSZ in alike biological microenvironments.

## Supplementary information

Supplementary Information
