# Supplementary material for: Probing the Solute-Solvent Interaction of an Azo-Bonded Prodrug in Neat and Binary Media: Combined Experimental and Computational Study
Source: Sci Rep. 2019 Feb 28;9:3023. doi: 10.1038/s41598-019-39028-1 (PMC6395765; doi:10.1038/s41598-019-39028-1)
Supplement: Supplementary file 1 — Supplementary Information [file 41598_2019_39028_MOESM1_ESM.docx]

| **Solvent** | **ε** | **n** | **∆ƒ** | **π*** | **α** | **β** | **λ_max2_ (nm)** |
| --- | --- | --- | --- | --- | --- | --- | --- |
| ***polar protic*** |  |  |  |  |  |  |  |
| water | 78.4 | 1.333 | 0.320 | 1.09 | 1.17 | 0.18 | 358 |
| methanol | 32.7 | 1.329 | 0.308 | 0.6 | 0.93 | 0.62 | 367 |
| ethanol | 24.6 | 1.361 | 0.289 | 0.54 | 0.83 | 0.77 | 367 |
| isopropanol | 19.9 | 1.378 | 0.276 | 0.48 | 0.76 | 0.95 | 358 |
| ***Polar aprotic*** |  |  |  |  |  |  |  |
| acetonitrile | 36.6 | 1.344 | 0.305 | 0.75 | 0.19 | 0.31 | 357 |
| ethylacetate | 6.1 | 1.372 | 0.201 | 0.55 | 0 | 0.45 | 356 |
| acetone | 20.6 | 1.359 | 0.284 | 0.71 | 0.08 | 0.48 | 358 |
| THF | 7.6 | 1.407 | 0.210 | 0.58 | 0 | 0.55 | 363 |
| ***nonpolar*** |  |  |  |  |  |  |  |
| chloroform | 4.8 | 1.446 | 0.148 | 0.58 | 0.44 | 0 | 358 |
| 1,4-Dioxane | 2.2 | 1.422 | 0.020 | 0.55 | 0 | 0.37 | 360 |

**Probing the Solute-Solvent Interaction of an Azo-Bonded Prodrug in Neat and Binary media: Combined Experimental and Computational study**

Abdulilah Dawoud Bani-Yaseen^*^, Amina S. Al-Jaber, Heba M. Ali

^(1)^ Department of Chemistry & Earth Sciences, College of Arts & Science, Qatar University, Doha, P.O. Box 2713, State of Qatar

**Supplementary Information**

**Table S1: solvent parameters and experimental data#**

# **ε**: solvent electric permittivity; **n**: refractive index; **∆ƒ**: orientation polarizability;

**π***: index of solvent’s dipolarizability; **α**: hydrogen bond donor acidity; **β**: hydrogen bond donor basicity


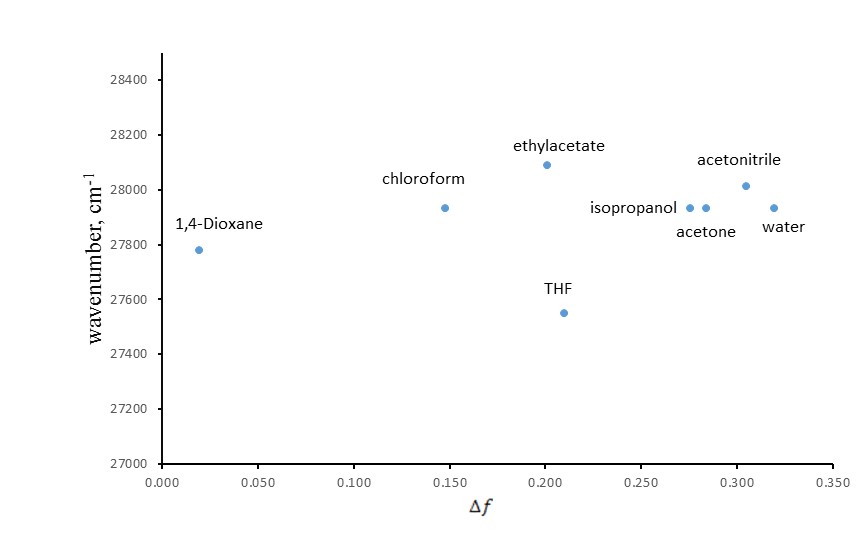


**Fig.S1 effect of solvent’s polarizability function (∆ƒ) on the maximum wavenumber of SSZ**


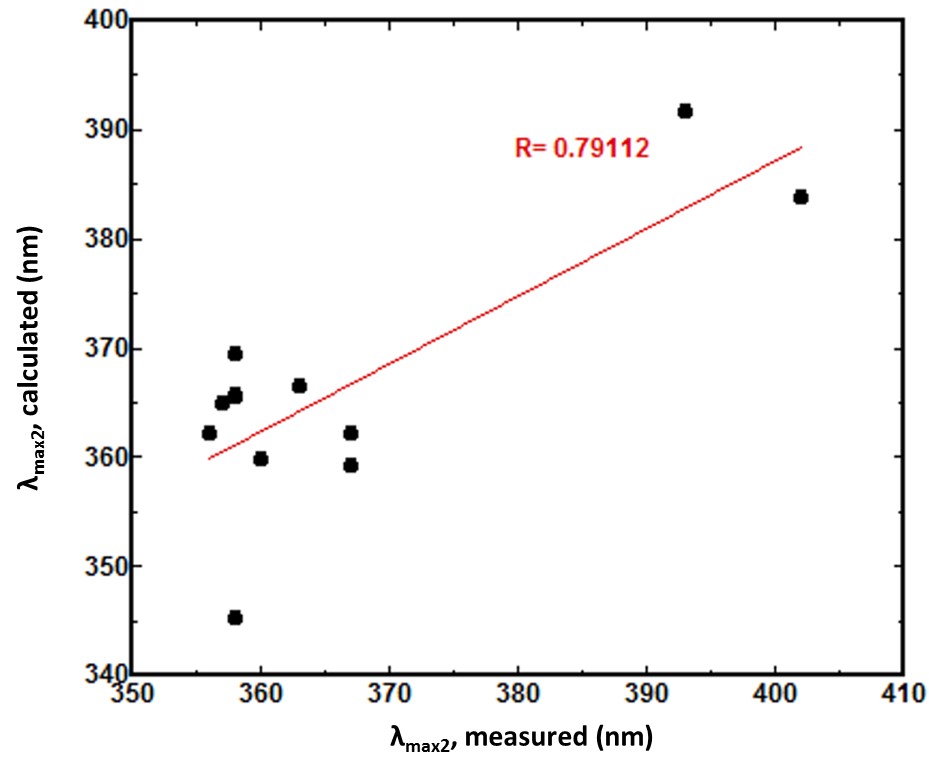


**Fig.S2 Comparison between measured and calculated λ_max_ SSZ in neat solvents**


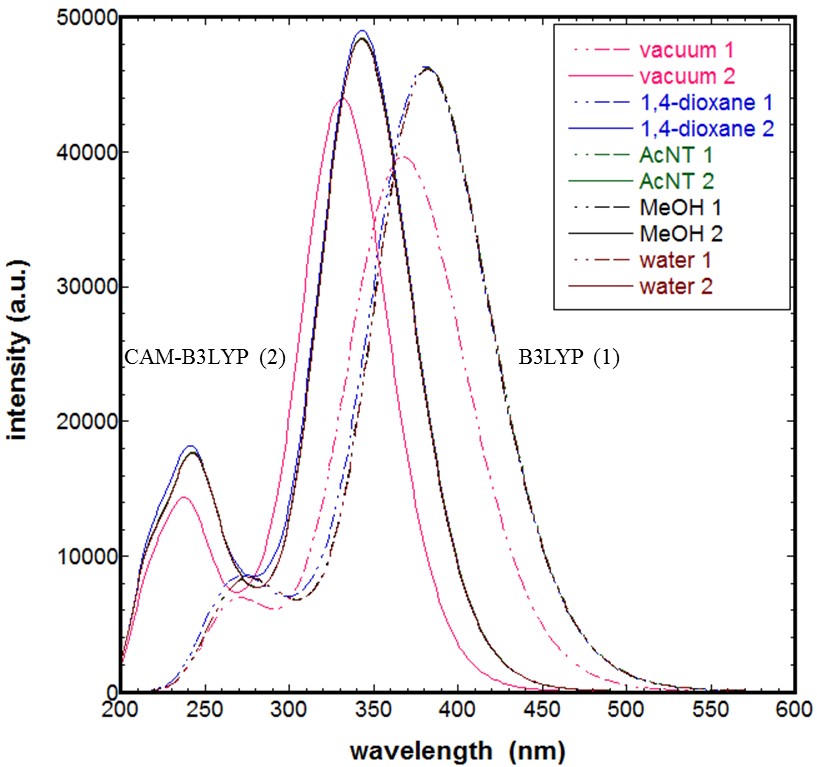


**Fig.S3 solvent effect on the simulated UV-Vis absorption spectra of SSZ.**

**Two functionals: B3LYP (1) and CAM-B3LYP (2)**

**
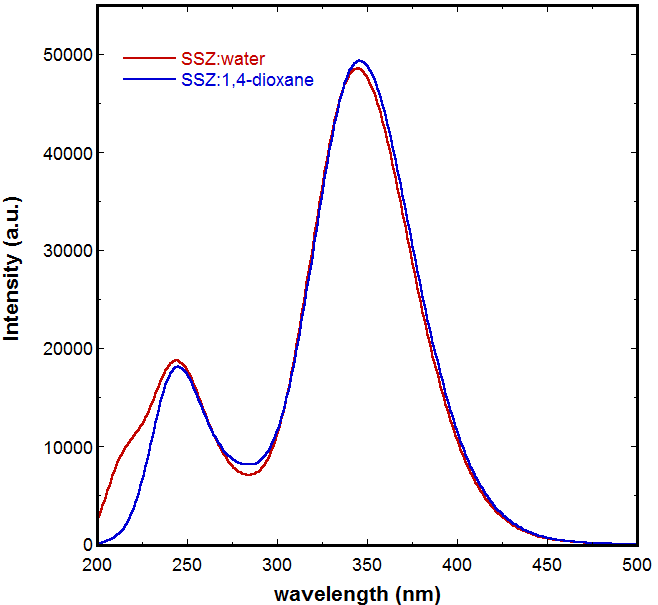
**

**Fig.S4 Absorption spectra of 1:2 HB complexes of SSZ:water and SSZ:1,4-dioxane; simulated using DFT/CAM-B3LYP/6-31G+(d)/IEFPCM methods**
